# The small molecule fenpropimorph rapidly converts chloroplast membrane lipids to triacylglycerols in *Chlamydomonas reinhardtii*

**DOI:** 10.3389/fmicb.2015.00054

**Published:** 2015-02-24

**Authors:** Hanul Kim, Sunghoon Jang, Sangwoo Kim, Yasuyo Yamaoka, Daewoong Hong, Won-Yong Song, Ikuo Nishida, Yonghua Li-Beisson, Youngsook Lee

**Affiliations:** ^1^Division of Molecular and Life Sciences, Pohang University of Science and TechnologyPohang, South Korea; ^2^Division of Life Science, Graduate School of Science and Engineering, Saitama UniversitySaitama, Japan; ^3^Department of Plant Biology and Environmental Microbiology, Commissariat à l’Énergie Atomique et aux Énergies Alternatives – Centre National de la Recherche Scientifique – Aix-Marseille UniversitySaint-Paul-Lez-Durance, France; ^4^POSTECH-UZH Global Research Laboratory, Division of Integrative Biology and Biotechnology, Pohang University of Science and TechnologyPohang, South Korea

**Keywords:** membrane lipid recycling, *Chlamydomonas reinhardtii* fenpropimorph, biofuel, triacylglycerol

## Abstract

Concern about global warming has prompted an intense interest in developing economical methods of producing biofuels. Microalgae provide a promising platform for biofuel production, because they accumulate high levels of lipids, and do not compete with food or feed sources. However, current methods of producing algal oil involve subjecting the microalgae to stress conditions, such as nitrogen deprivation, and are prohibitively expensive. Here, we report that the fungicide fenpropimorph rapidly causes high levels of neutral lipids to accumulate in *Chlamydomonas reinhardtii* cells. When treated with fenpropimorph (10 μg mL^-1^) for 1 h, *Chlamydomonas* cells accumulated at least fourfold the amount of triacylglycerols (TAGs) present in the untreated control cells. Furthermore, the quantity of TAGs present after 1 h of fenpropimorph treatment was over twofold higher than that formed after 9 days of nitrogen starvation in medium with no acetate supplement. Biochemical analysis of lipids revealed that the accumulated TAGs were derived mainly from chloroplast polar membrane lipids. Such a conversion of chloroplast polar lipids to TAGs is desirable for biodiesel production, because polar lipids are usually removed during the biodiesel production process. Thus, our data exemplified that a cost and time effective method of producing TAGs is possible using fenpropimorph or similar drugs.

## INTRODUCTION

Glycerolipids are ubiquitous in all cell types. Membrane lipids consist mainly of polar glycerolipids, which assemble into a bilayer structure that delineates the boundary of cells and provides sites of interaction for many proteins. Storage lipids are mainly neutral glycerolipids, including triacylglycerols (TAGs). They are stored in lipid droplets (LDs) in the seeds of plants, adipose cells of animals, and in algal cells. Polar membrane lipids and TAGs share some common biosynthetic pathways and have a common precursor, i.e., diacylglycerols (DAGs). Under stress conditions, membrane lipids are degraded, and the released acyl chains or DAG backbones can be re-assembled into neutral lipids, and stored in LDs, which are a major energy source for re-growth when conditions turn favorable (Hu et al., 2008; Siaut et al., 2011). TAGs are highly reduced, energy-rich compounds that can provide energy for humans, livestock, and industry. The rapid conversion of membrane lipids into TAGs is industrially beneficial, because TAGs are a more efficient and cost-effective source of energy than are polar lipids and they are more readily converted into diesel.

Global warming and climate change, which are thought to result from the extensive use of fossil fuels and the consequent increase in carbon dioxide levels in the air, threaten the lives of humankind and many other organisms. Energy sources that do not increase atmospheric carbon dioxide levels, such as biodiesel, are in high demand, and photosynthetic organisms are being intensively studied as potential clean, sustainable, and renewable energy sources. Most biofuels developed to date are derived from carbohydrates from *Saccharum* (sugarcane) and *Solanum tuberosum* (potato) or from TAGs from plants such as *Elaeis guineensis* (oil palm), *Brassica napus* (canola), *Olea europaea* (olive), *Helianthus annuus* (sunflower), and *Zea mays* (maize). However, these oil-producing plants are also important sources of food and feed, thus raising an ethical argument against using such plants as energy sources (Hill et al., 2006).

During the past decade, algae have emerged as an alternative non-crop source for biofuel, because they (i) do not compete with food-providing plants for agricultural land use, (ii) some species can accumulate large amounts of lipids that can be used for biodiesel production (for example, some microalgae can accumulate up to 50% of their biomass as oils; Tornabene et al., 1983; Miao and Wu, 2006; Xu et al., 2006), and (iii) grow very fast, fixing solar energy with an efficiency that is about 10–20% higher than that of land plants (Li et al., 2008). However, despite these advantages, several technological obstacles need to be overcome before it becomes economically feasible to culture microalgae for biofuel production. For example, microalgae accumulate massive amounts of oil when subjected to stress conditions such as nitrogen deprivation. However, it is time-consuming and costly to induce microalgal lipid accumulation through nitrogen starvation.

*Chlamydomonas reinhardtii* has been widely used as a model organism to investigate various microalgal processes, including lipid metabolism (Merchant et al., 2012; Liu and Benning, 2013). Furthermore, this alga displays a sexual reproduction cycle that allows genetic analysis of phenotypes. *Chlamydomonas* accumulates high levels of TAGs in LDs under stress conditions such as nutrient deficiency or exposure to high intensity light (Miller et al., 2010; Fan et al., 2011). As in terrestrial plants, two types of enzymes participate in the final step of TAG synthesis in *Chlamydomonas*, i.e., diacylglycerol acyltransferases (DGATs) and phospholipid:diacylglycerol acyltransferases (PDATs). These enzymes catalyze the formation of TAG from a DAG molecule. Genes encoding DGAT proteins in *Chlamydomonas* are strongly induced under TAG-accumulating conditions, such as nitrogen or other nutrient deprivation (Boyle et al., 2012). The PDAT in *Chlamydomonas* has been demonstrated *in vitro* to use phospholipids and galactolipids as acyl donors, and DAG as acyl acceptors (Yoon et al., 2012). Insertional mutants lacking PDAT accumulated 25% less oil than its wild-type progenitor, demonstrating the importance of the PDAT-mediated acyl-remodeling pathway in oil accumulation in *Chlamydomonas* (Boyle et al., 2012).

Altering sterol levels was reported to affect sterol and fatty acid metabolism in fungal and animal cells (Colgan et al., 2007), and to result in the cleavage of ER membrane-bound transcription factors by membrane-associated proteases, and thereby to activate transcription factors in animal cells (Sakai et al., 1998; Porter et al., 2010). The activated transcription factors move to the nucleus, where they up-regulate the expression of genes involved in the biosynthesis of sterols and fatty acids (Horton et al., 2002; Porter et al., 2010). If such a mechanism also exists in microalgae, it might represent a means of manipulating lipid accumulation. However, it is not known how changes in sterol levels affect oil metabolism in microalgae. Sterol metabolism can be altered by chemical treatment (Ryder et al., 1986; Campagnac et al., 2008). For instance, fenpropimorph is an anti-fungal chemical that inhibits Δ^14^-reduction and/or Δ^8^–Δ^7^ isomerization in the sterol biosynthetic pathway (Campagnac et al., 2008). This chemical effectively inhibits sterol biosynthesis and alters the composition of sterols in fungi, yeasts, and plants (Baloch et al., 1984; Ziogas et al., 1991; Debieu et al., 1992; Moebius et al., 1996; Hartmann et al., 2002; Campagnac et al., 2009). Leek seedlings grown in the presence of fenpropimorph for 7 days have decreased total sterol levels, and accumulate LDs in the roots (Hartmann et al., 2002).

Here, we report that treatment of *C. reinhardtii* with fenpropimorph very rapidly induces the formation of LDs filled with TAGs. Surprisingly, this effect is not accompanied by any changes in sterol metabolism, but appears to induce the conversion of monogalactosyldiacylglycerol (MGDG), a plastidial polar lipid, to TAGs. In addition, the drug induces cell death and cell precipitation, which might facilitate the harvesting of TAGs in an industrial setting. Thus the treatment of algae with fenpropimorph results in three favorable changes for biodiesel production: a rapid increase in cellular TAG levels; a decrease in polar lipid level *in vivo*; and the efficient precipitation of algal cells.

## MATERIALS AND METHODS

### CELL CULTURE AND MEASUREMENT OF CELL CONCENTRATION

The *C. reinhardtii* strain CC-125 wild type *mt*^+^ (137c) was obtained from the Chlamydomonas Genetics Center (USA). *Chlamydomonas* cells were cultured in Tris-acetate phosphate (TAP) medium (Rippka et al., 1979) or in the same medium without acetate (for photoautotrophic culture) at 25°C under continuous light (25–30 μmol photons m^-2^ s^-1^) while shaking at 160 rpm. Cell growth was monitored by measuring the optical density (OD) at 750 nm using a Safire fluorescence spectrophotometer (TECAN, Switzerland) and also by counting the cell number using a hemocytometer.

### ESTIMATION OF CELL SURFACE AREA CHANGE

The areas of plastids and entire cells in the photographs of the cells were measured using ImageJ (Papadopulos et al., 2007), to estimate the changes in their surface area.

### CHEMICAL TREATMENT, LD STAINING, AND QUANTIFICATION OF FLUORESCENCE INTENSITY (FI)

A stock solution of fenpropimorph (SANTA CRUZ, cat#SC-235130) was prepared in pure ethanol at a concentration of 10 mg mL^-1^ and stored at room temperature. *Chlamydomonas* cells at late mid-log phase of growth were treated by adding fenpropimorph to a final concentration of 10 μg mL^-1^ and incubated for up to 1 h under standard growth conditions in TAP medium containing acetate and nitrogen sources, except when otherwise noted. To visualize the LDs, fenpropimorph-treated cells were stained with Nile red at a final concentration of 1 μg mL^-1^ (prepared from a stock solution of 0.1 mg mL^-1^ in acetone) for 30 min in the dark at room temperature (∼25°C; Kim et al., 2013). To quantify the FI of LDs, fenpropimorph-treated cells were dispensed into a 96-well plate (Tissue culture testplate, SPL) and stained with Nile red, and the fluorescence signals from LDs were measured using a Safire fluorescence spectrophotometer (TECAN, Switzerland) with a 488 nm excitation filter and a 565 nm emission filter. Stained cells were also observed under fluorescence microscopy (Ziess, Axioskop 2 MOT, Germany) using filter set 44 (excitation BP 475/40, emission BP 530/50).

### LIPID EXTRACTION AND ANALYSES

*Chlamydomonas* CC-125 cells at late mid-log phase (approximately 6.0 × 10^6^ cells mL^-1^) were treated either with 10 μg mL^-1^ of fenpropimorph for 1 h or with ethanol alone (solvent control). Cells were harvested by centrifugation at 2,000 g for 5 min. Lipid extraction and analyses were conducted following the methods described in Kim et al. (2013).

### STEROL ANALYSIS

For the quantification of sterols, [25,26,26,26,27,27,27-^2^H_7_] cholesterol (AVANTI cat# 700116) was added to the total lipid extract as an internal standard. Sterol analysis was performed as previously described (Suzuki et al., 2004). Sterols were identified by comparison of their mass-spectra patterns with those of the published mass spectra patterns on known sterols (Miller et al., 2012).

### RADIOISOTOPE LABELING EXPERIMENT FOR DIACYLGLYCEROL (DAG) DETECTION

*Chlamydomonas* strain CC-125 cells were cultured to late mid-log phase (∼6 × 10^6^ cells mL^-1^) in TAP medium. Cells were labeled with 0.2 μCi mL^-1^ of [^14^C]acetate for 2 h, washed twice with acetate free TAP medium, and then treated with fenpropimorph at a concentration of 10 μg mL^-1^ for 1 h. Total lipids were extracted using chloroform/methanol/formic acid (10:10:1, by volume; Härtel et al., 2000). After centrifugation (2000 *g*; 10 min), the bottom phase was directly loaded onto a thin-layer chromatography (TLC) plate (TLC Silica gel 60, MERCK) and separated using a two-phase solvent mixture; with a first phase of [chloroform/methanol/acetic acid/water, 90/15/10/3 by volume] and a second phase of [hexane/diethyl ether/acetic acid, 80/30/1 by volume]. Neutral lipid separation was performed using the first phase solvent mixture. After completely drying the TLC plate, polar lipids were separated using the second phase solvent mixture. The plate was then exposed to an imaging plate (BAS-TR 2040S, Fujifilm) and the relative strength of the radioactivity of [^14^C]acetate was visualized with an image analyzer (FLA-2000, Fujifilm). The neutral lipid spots containing TAG and DAG, and polar lipid spots were then scraped off the plate and recovered separately, and the radioactivity of each spot was determined by liquid scintillation counting (Tri-Carb 2910 TR, Perkin Elmer).

## RESULTS

### FENPROPIMORPH INDUCES TAG ACCUMULATION IN *C. reinhardtii*

Small chemical molecules are known to trigger metabolic changes, as has been demonstrated in many cell systems. In our search for an efficient and alternative method to trigger oil accumulation in microalgae, we tested the hypothesis that fenpropimorph can induce cellular changes in microalgal oil content. This hypothesis was based on the observation that fenpropimorph disturbs sterol homeostasis in other organisms, which is correlated with ER stress response, changes in fatty acid and lipid metabolism (Baloch et al., 1984; Ziogas et al., 1991; Debieu et al., 1992; Moebius et al., 1996; Hartmann et al., 2002; Campagnac et al., 2009).

**FIGURE 1:**
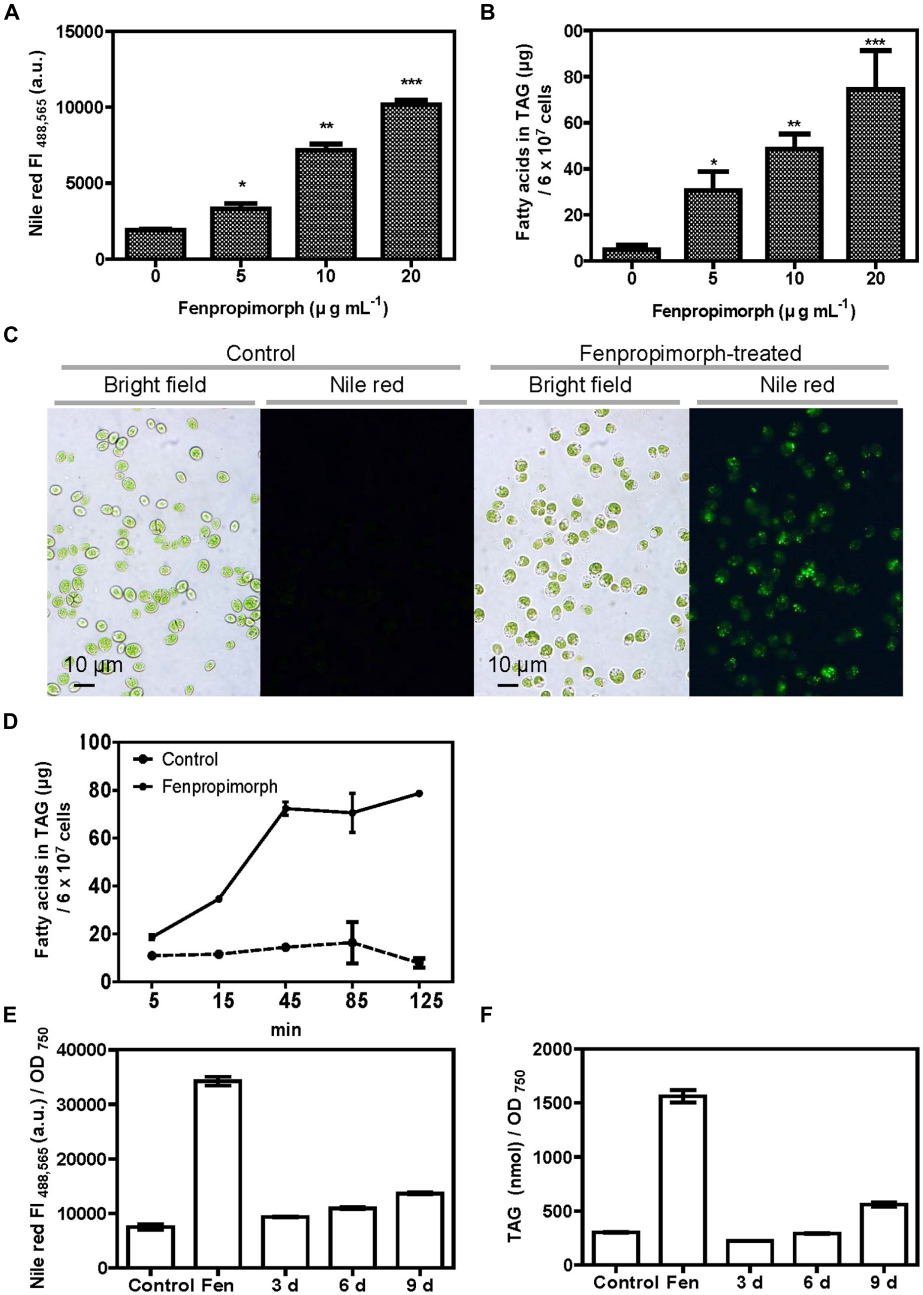
**Fenpropimorph induces neutral lipid accumulation in *Chlamydomonas reinhardtii*. (A)** Fenpropimorph-induced LD formation occurs in a dose-dependent manner. The fluorescence intensity (FI) of a neutral lipid specific-dye, Nile red, was determined. Late mid-log phase *Chlamydomonas* cells (N+, acetate+) were treated with ethanol (solvent control) or fenpropimorph (1 h, at RT). Averages from three replicate experiments are presented. Bars represent SE. Significant differences, as determined by Student’s *t*-test, are indicated by asterisks (*p < 0.05, **p < 0.01, ***p < 0.001). **(B)** Fenpropimorph-induced TAGs were extracted and analyzed using biochemical methods. Control cells were treated with the same volume of ethanol used to dissolve fenpropimorph. Averages from triplicate experiments are presented. Bars represent SE. Significant differences, as determined by Student’s *t*-test, are indicated by asterisks (*p < 0.05, **p < 0.01, ***p < 0.001). **(C)** Images of Nile red-stained LD accumulation in fenpropimorph-treated cells. Cells were treated with fenpropimorph for 1 h. Images were obtained using a fluorescence microscope. **(D)** Time-dependent change in TAG concentration in fenpropimorph-treated *Chlamydomonas* cells. TAG accumulation induced by fenpropimorph (10 μg mL^-1^) treatment was analyzed biochemically. Averages and SE from three replicate experiments are presented. TAG levels shown were converted to μg from nmol values obtained from GC experiment. The original nmol values for each time point (5, 15, 45, 85, and 125 min) were 26.8 ± 4.7, 40.9 ± 0.4, 51.2 ± 5.1, 66.1 ± 24.2, and 27.7 ± 5.7, respectively, for control samples, and 65.4 ± 3.0, 120.7 ± 1.2, 255.0 ± 8.5, 260.7 ± 21.9, and 278.0 ± 1.0, respectively, for fenpropimorph-treated samples. In experiments shown in **(A – D)**, *Chlamydomonas* cells in late mid-log phase culture in TAP medium (N+, acetate+) were used. **(E,F)** Comparison of the effect of nitrogen deprivation and fenpropimorph treatment on lipid induction efficiency in *Chlamydomonas* cells. **(E)** Nile red fluorescence intensity of control *Chlamydomonas* cells, and of cells subjected to fenpropimorph treatment (1 h, 25°C), and nitrogen deprivation (for the indicated number of days). *Chlamydomonas* cells were grown in normal conditions to mid-log phase, and washed to remove acetate and nitrogen from the medium. They were then re-suspended in TAP medium without an acetate or nitrogen source, and then either treated with ethanol (solvent control) or fenpropimorph (10 μg mL^-1^) for 1 h, or transferred to the nitrogen-deficient conditions and incubated for 3, 6, or 9 days. The FI value was measured. Averages from three replicate experiments are presented. Bars represent SE. **(F)** Biochemical analysis of TAG content in cells treated as in **(E)**. Averages from three replicate experiments are presented. Bars represent SE.

**Figure A1:**
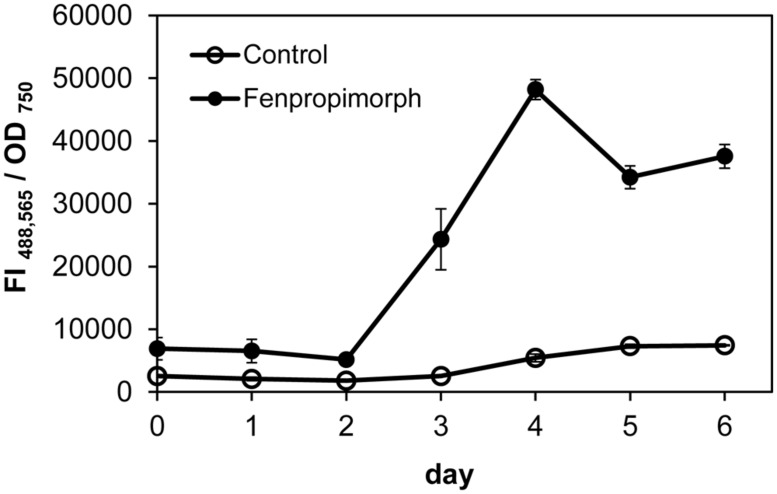
**Growth phase-dependent effect of treatment with fenpropimorph (10 μg mL^–1^) on Nile red fluorescence of *Chlamydomonas*.** The fluorescence values are normalized by optical density at 750 nm. Averages from three experimental replicates are presented. Bars represent SE.

*Chlamydomonas* cells were treated with 10 μg mL^-1^ of fenpropimorph, and the cellular oil content was tracked by monitoring the fluorescence intensity (FI) of Nile red, a lipophilic dye specific for neutral lipids (Kou et al., 2013). After just 1 h of fenpropimorph treatment, the FI increased dramatically (**Figure 1A**). Furthermore, the FI increased as the concentrations of fenpropimorph increased from 5 to 20 μg mL^-1^ (**Figure 1A**). Biochemical analyses of lipids extracted from fenpropimorph-treated cells revealed that TAGs increased 6–15-fold in fenpropimorph-treated versus control *Chlamydomonas* cells, in proportion to the increases in drug concentrations from 5 to 20 μg mL^-1^ (**Figure 1B**). The fold increase values varied between experiments, perhaps due to slight differences in culture age (within the late mid-log phase), and consequent variation in the TAG levels of the control samples which ranged between 40 and 135 nmol fatty acids in TAG per 6 × 10^7^cells. The effect of the drug was maximal at the late mid-log phase (at 4 days of culture under our conditions), and slightly less at the stationary phase (**Figure A1**). Microscopic observations revealed that fenpropimorph-treated cells had more LDs than did the control cells (**Figure 1C**). The effect of fenpropimorph treatment on TAG induction in *Chlamydomonas* was very rapid: TAG levels increased significantly after as little as 5 min of treatment with 10 μg mL^-1^ fenpropimorph, and became saturated at 45–85 min (i.e., longer treatment did not induce further TAG accumulation; **Figure 1D**).

### FENPROPIMORPH INDUCES THE FORMATION OF MORE TAGS THAN DOES UP TO 9 DAYS OF N STARVATION IN MEDIUM WITH NO ACETATE SUPPLEMENT

Since nitrogen starvation is the most extensively studied trigger of oil accumulation in microalgae, we compared the amount of TAGs induced by fenpropimorph to that induced by N starvation. *Chlamydomonas* cells were grown in normal conditions to mid-log phase, washed to remove acetate and nitrogen from the medium, then re-suspended in TAP medium without an acetate or nitrogen source. We did not include acetate in the medium in this experiment since the biofuel industry often does not use acetate in the medium to avoid additional cost. Nile red FI value and TAG amount of cells treated with fenpropimorph for 1 h was found to be ∼2.5-fold higher than those cells starved of N for up to 9 days (**Figures 1E,F**).

**FIGURE A2:**
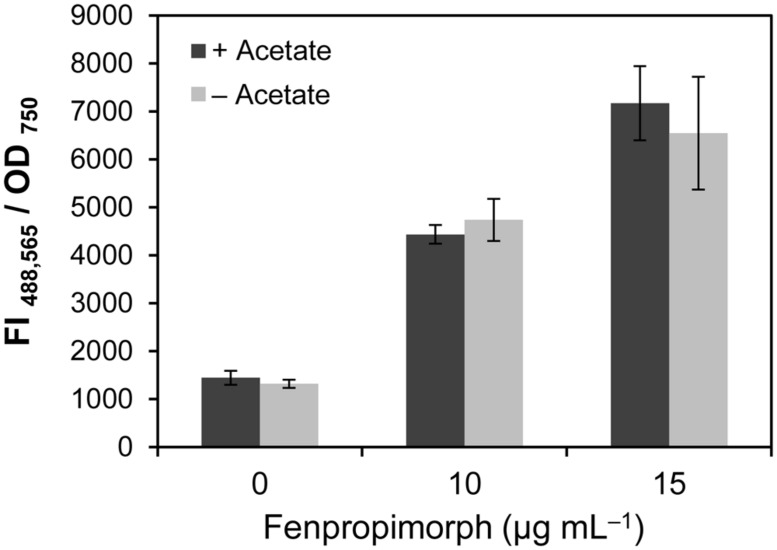
**Acetate in the medium was not necessary for the fenpropimorph effect on TAG accumulation.** Note the absence of difference in Nile red fluorescence intensities of cells treated with fenpropimorph in media with or without acetate. *Chlamydomonas* cells in late mid-log phase culture in the TAP (N+, acetate+) medium were harvested, and resuspended either in the same TAP medium or in TAP medium without acetate, then treated with fenpropimorph (10 or 15 μg mL^-1^) for 1 h at 25°C. Averages from three experimental replicates are presented. Bars represent SE.

We then tested whether the effect of fenpropimorph was dependent on the presence of acetate in the medium. Interestingly, the presence of acetate in medium did not make any significant difference in Nile red fluorescence values of *Chlamydomonas* cells treated with 10 or 15 μg mL^-1^ fenpropimorph (**Figure A2**).

### FENPROPIMORPH INCREASES TAG CONTENT BY REMODELING PLASTIDIAL LIPIDS

**FIGURE 2:**
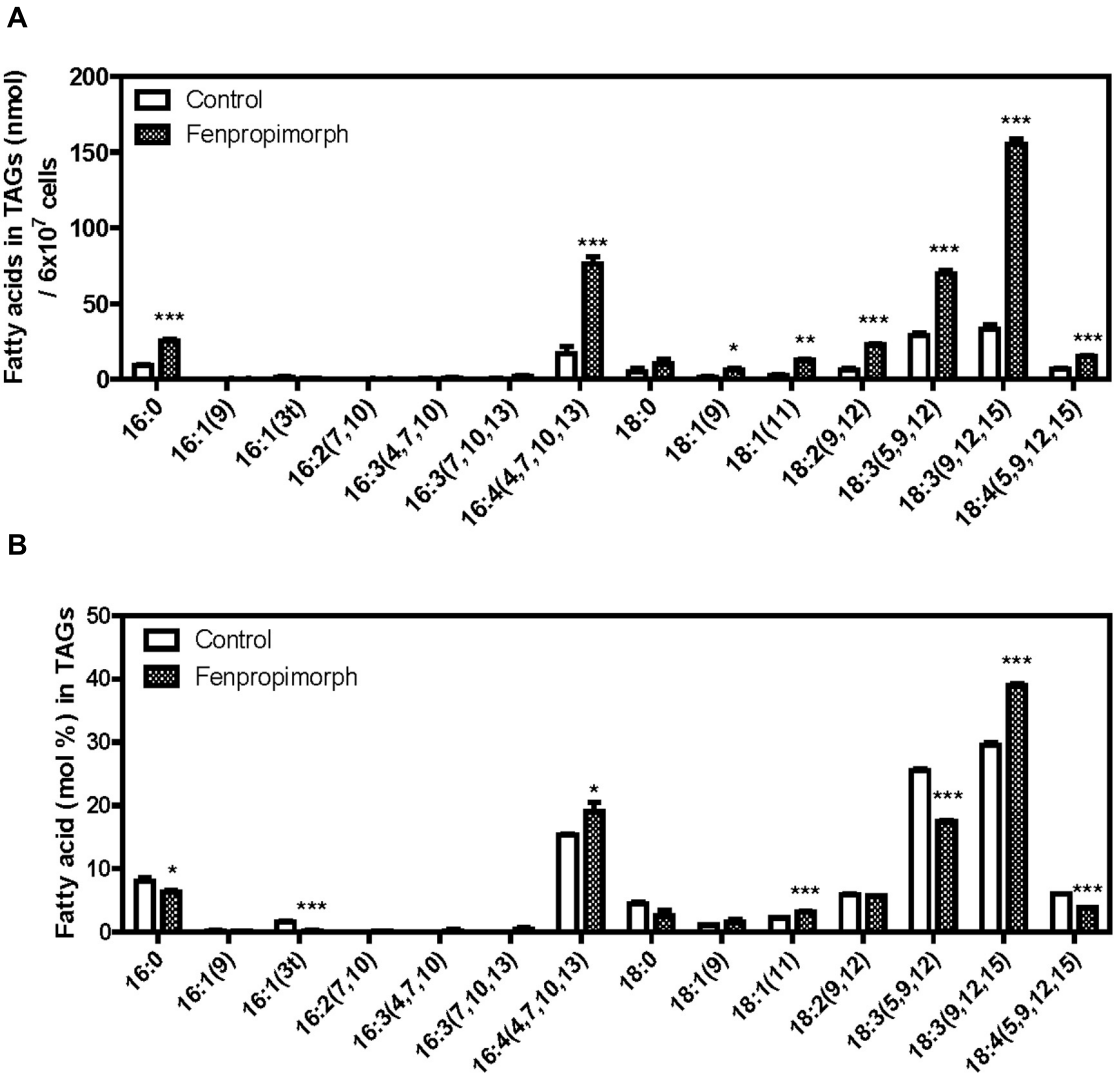
**Biochemical analysis of TAGs in fenpropimorph-treated *C. reinhardtii* in late mid-log phase culture in TAP medium (N+, acetate). (A)** A comparison of the absolute amount of fatty acids in TAGs isolated from fenpropimorph-treated and control cells. Averages from three replicate experiments are presented. Bars represent SE. Significant differences, as determined by Student’s *t*-test, are indicated by asterisks (*p < 0.05, **p < 0.01, ***p < 0.001). **(B)** Comparison of fatty acid mol% in TAGs isolated from fenpropimorph-treated cells and control cells. Averages from three replicate experiments are presented. Bars represent SE. Significant differences, as determined by Student’s *t*-test, are indicated by asterisks (*p < 0.05, **p < 0.01, ***p < 0.001).

To identify the biosynthetic origin of the fatty acids accumulated in the TAG fraction in fenpropimorph-treated cells, the fatty acid content and composition of the TAG fraction were compared between control and fenpropimorph-treated cells (**Figures 2A,B**). All fatty acid levels in TAG increased in fenpropimorph-treated cells, but the highest increases were found in 16:4(4,7,10,13) and 18:3(9,12,15) (**Figure 2A**), the two fatty acids that preferentially occur in plastidial lipids (Giroud et al., 1988; Fan et al., 2011). Fatty acid compositional analysis (mol% values) confirmed the preferential accumulation of plastidial-type fatty acids in TAGs induced by fenpropimorph (**Figure 2B**). These results suggest that the fatty acids formed in response to fenpropimorph treatment are most likely derived from recycled plastidial lipids.

**FIGURE 3:**
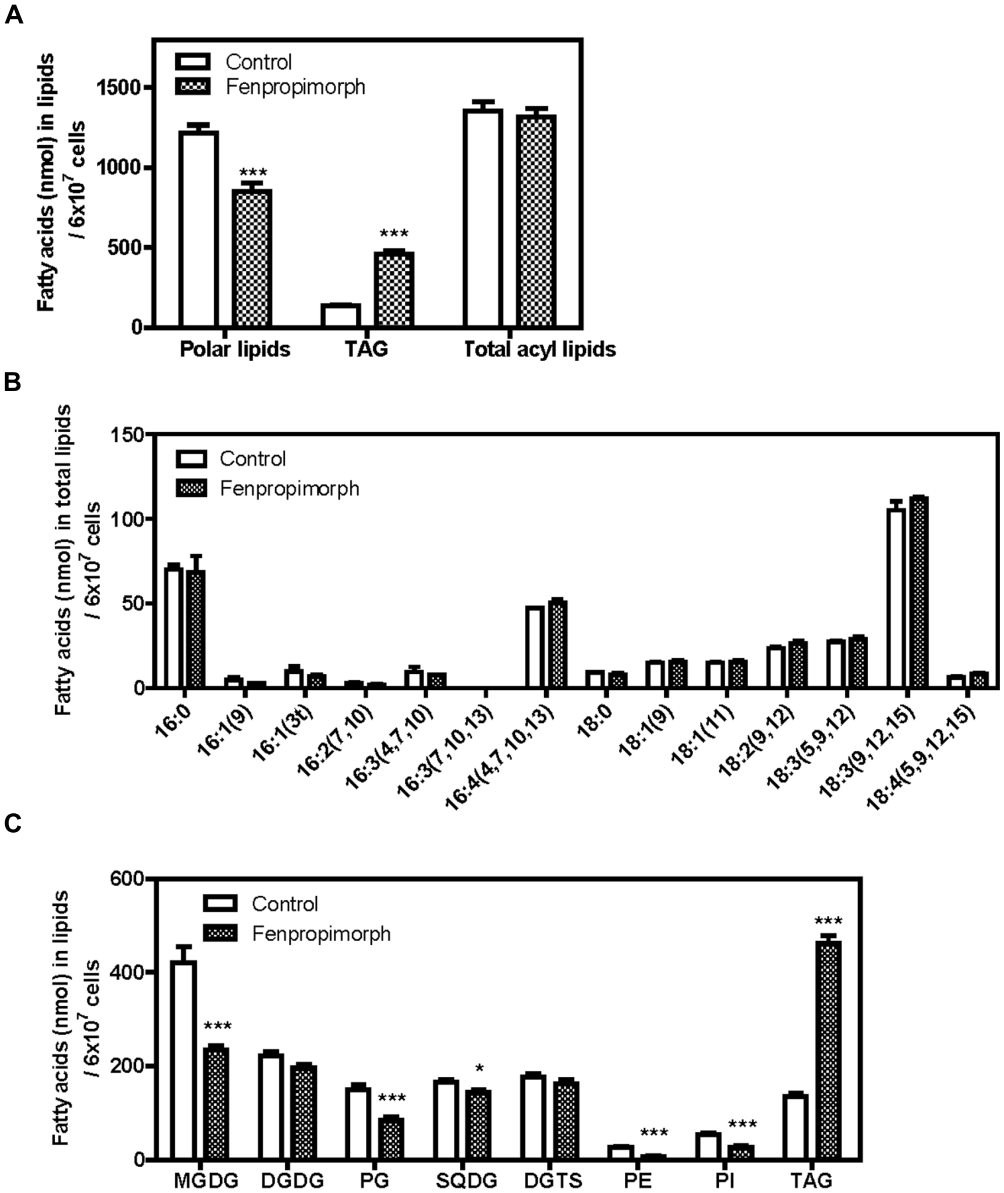
**Alterations in polar membrane lipid and TAG profiles of fenpropimorph-treated *C. reinhardtii* at late mid-log phase cultured in TAP medium (N+, acetate+). (A)** Comparison of the total fatty acid amount in polar lipids, TAGs and total acyl lipids. Bars represent SE (*N* = 2, *n* = 3). Significant differences, as determined by Student’s *t*-test, are indicated by asterisks (***p < 0.001). **(B)** Comparison of fatty acid profiles in total lipids. A representative result of 10 experiments is shown. Bars represent SE (*n* = 3). **(C)** Comparison of the concentration of major membrane lipids and TAGs between fenpropimorph-treated and control cells. Averages from three replicate experiments are presented. Bars represent SE (*N* = 2, *n* = 6). Significant differences, as determined by Student’s *t*-test, are indicated by asterisks (*p < 0.05, ***p < 0.001).

To examine this possibility, we analyzed the total acyl lipid contents and individual polar membrane lipids of *Chlamydomonas*. The decrease in polar lipids mirrored the increase in TAG levels (**Figure 3A**), and the total acyl-lipid contents did not differ between the control and the chemical-treated cells (**Figure 3A**, right), suggesting that fenpropimorph most likely induced remodeling of lipids. The fatty acid composition of total lipids (**Figure 3B**) did not differ between control and fenpropimorph-treated cells, thus further supporting this possibility.

**FIGURE A3:**
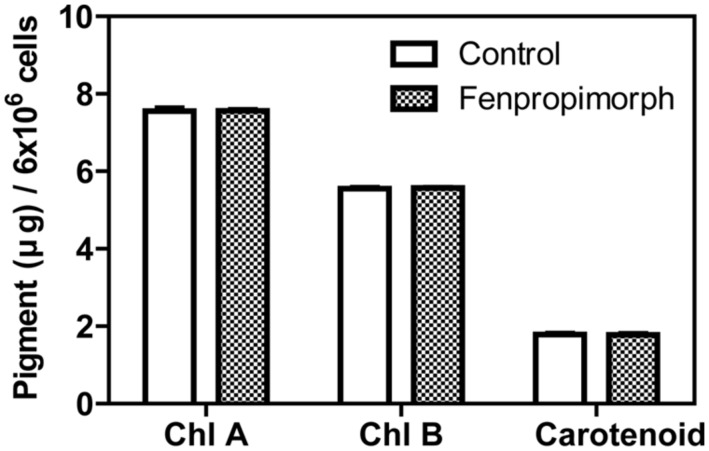
**Chlorophyll a and b and carotenoid contents of fenpropimorph-treated *Chlamydomonas* strain CC-125 cells.** Cells in TAP (N+, acetate+) medium were treated with fenpropimorph (10 μg mL^-1^) or ethanol (solvent control) for 1 h at 25°C. Chlorophyll and carotenoid contents were assayed following the method previously described (Lichtenthaler and Babani, 2004). Approximately 6.0 × 10^6^ cells were collected by centrifugation (2000 *g*; 10 min), and the pellet was resuspended in 95% ethanol and mixed by vortexing. After centrifugation, the supernatant was transferred to a new 96-well plate and optical absorbance at 648 nm and 664 nm were measured using a Safire fluorescence spectrophotometer (TECAN, Switzerland). Each pigment content was calculated as described previously (Lichtenthaler and Babani, 2004). Averages and SE from three replicate experiments are presented.

However, not all polar lipids changed to the same extent. The amounts of the most abundant plastidial galactolipids, MGDG, plastidial phospholipid phosphatidylglycerol (PG), and plastidial sulfur lipid sulfoquinovosyldiacylglycerol (SQDG) decreased to 56%, 57%, and 87% of those in untreated cells (**Figure 3C**). Compared to the above-mentioned plastidial lipids, the contribution of other membrane lipids seemed to be relatively small: the concentration of digalactosyldiacylglycerol (DGDG) changed to a much lesser extent than did that of MGDG, the diacylglyceryltrimethylhomoserine (DGTS) level did not change, and phosphatidylethanolamine (PE) and phosphatidylinositol (PI) levels were low even in the control cells (**Figure 3C**). These results are consistent with our view that the TAGs induced by fenpropimorph originate mainly from plastids, and suggest that MGDG is the major source of the fatty acids in TAGs induced by the chemical treatment. Interestingly, the levels of three pigments abundant in photosynthetic organisms were not altered (**Figure A3**), suggesting that MGDG is substantially degraded in response to the addition of fenpropimorph.

**FIGURE 4:**
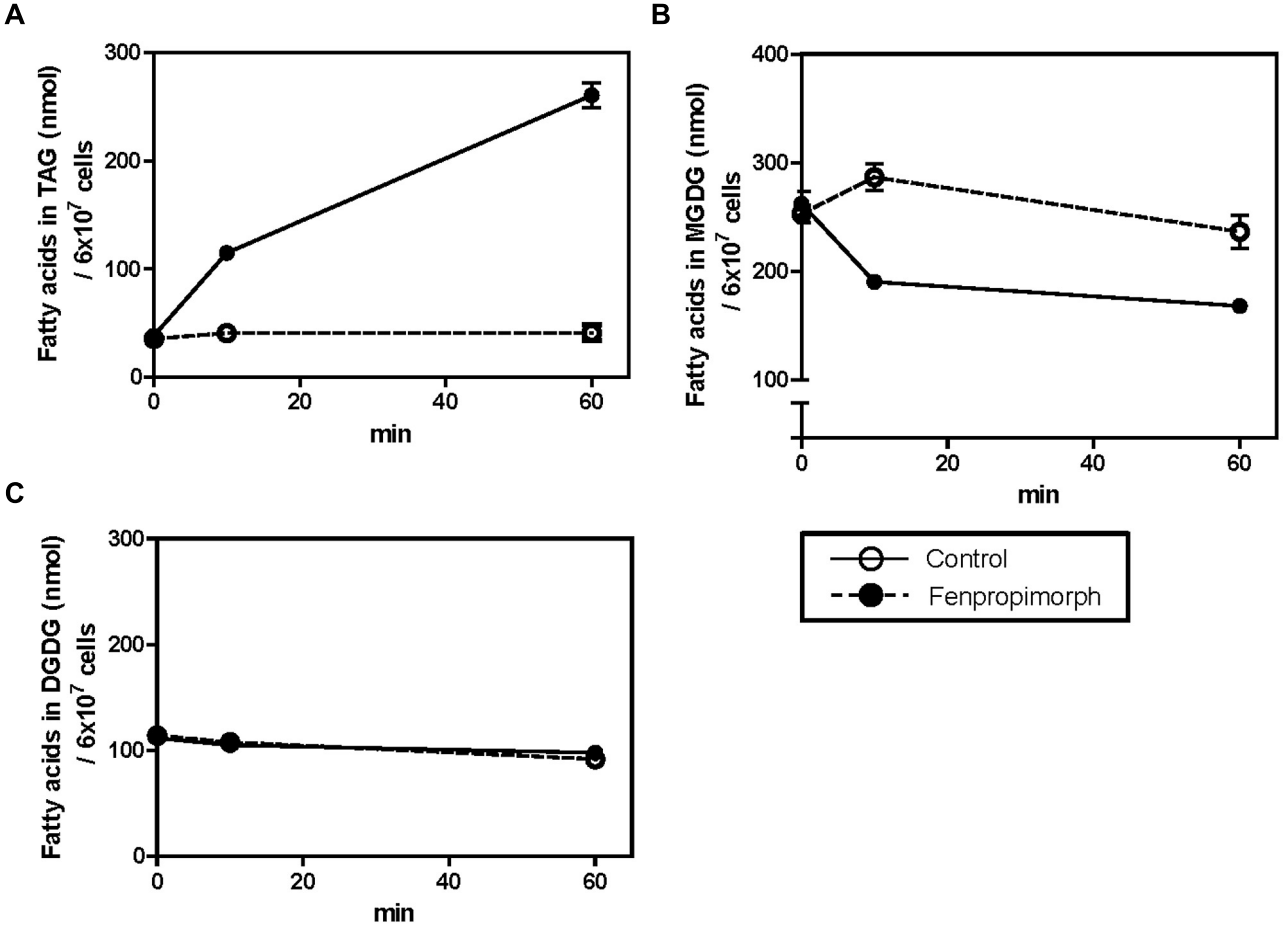
**Time-dependent changes in TAG **(A)**, MGDG **(B)**, and DGDG **(C)** contents in fenpropimorph-treated *C. reinhardtii*.** The cells were treated with 10 μg mL^-1^ fenpropimorph for up to 1 h at 25°C. *Chlamydomonas* cells in late mid-log phase culture in TAP medium (N+, acetate+) were used. Averages from three replicate experiments are represented. Bars represent SE.

If MGDG provides the major source of fatty acids in TAGs that accumulate under fenpropimorph treatment, then the time course of the increase in the level of TAGs and the decrease in the level of MGDG should exhibit an inverse relationship. To test this possibility, we determined the content of TAG and MGDG at three time points (after 0, 10, and 60 min of chemical treatment). As shown in **Figure 4**, the level of TAGs showed a 2.8-fold change at 10 min and at the 60 min, and a 6.4-fold increase compared to the mock-treatment (**Figure 4A**). The change in MGDG level was the opposite to that of TAGs at 10 min; the level of MGDG decreased to 66% in the chemical-treated cells compared to mock-treated cells. However, it did not decrease much further, and after 60 min of the drug treatment, the level of MGDG in the drug-treated cells remained at 71% of that of the control (**Figure 4B**). The DGDG level was not affected by the drug treatment (**Figure 4C**), as found in our previous experiment (**Figure 3C**). The results shown in **Figures 3 and 4** indicate that MGDG was an important source of FAs in TAGs accumulated by fenpropimorph treatment, especially at the beginning of the response to the drug, and other polar plastidial lipids PG and SQDG, and other polar lipids PE and PI, also contributed small portions.

**FIGURE 5:**
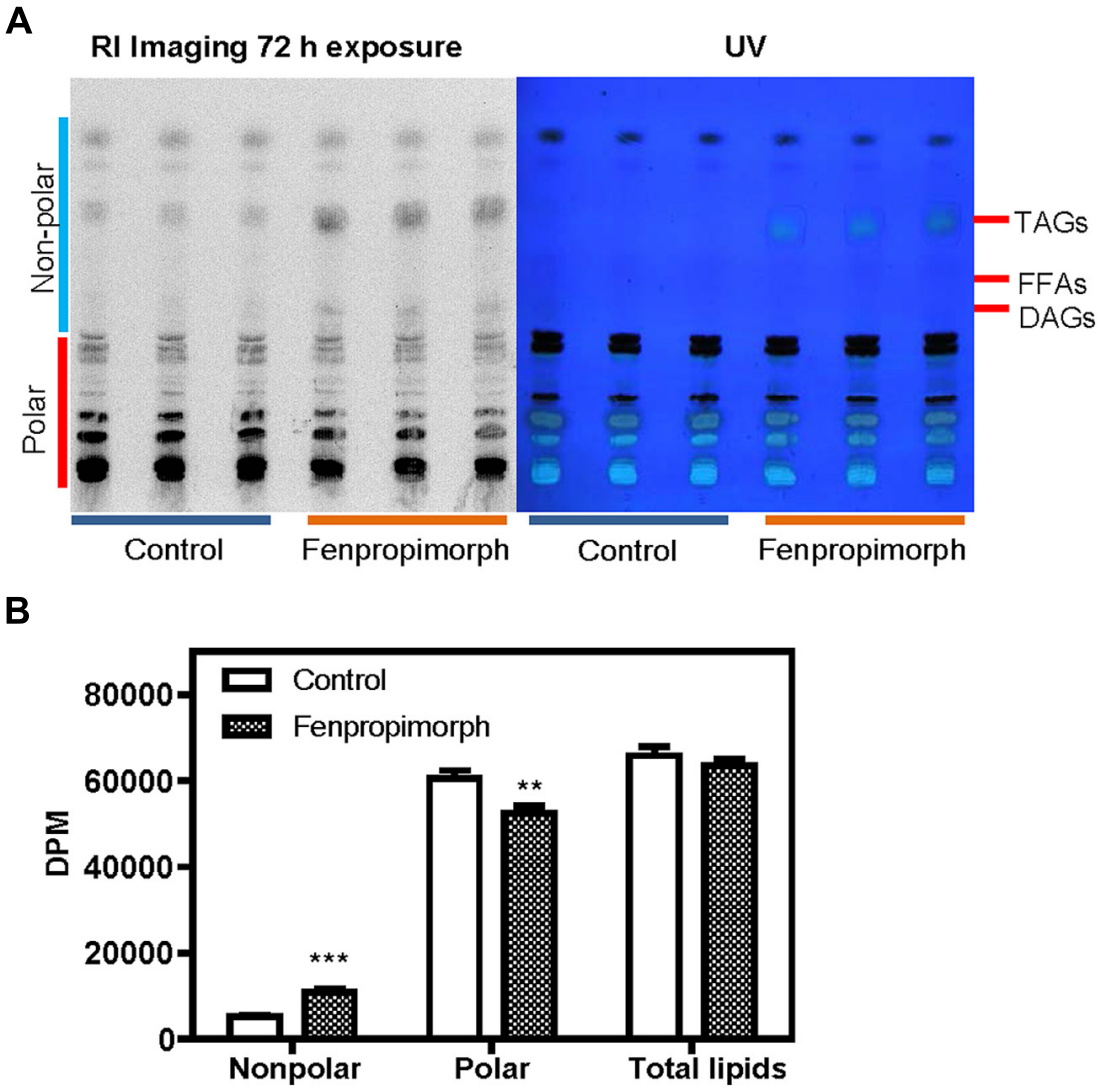
**Analysis of lipids labeled with [^**14**^C]acetate in fenpropimorph-treated *C. reinhardtii*. (A)** DAG is found to contain 14C-labeled in the fenpropimorph-treated cells. Two-phase TLC was performed as described in Materials and methods. The left image represents the radioactivity of lipids derived from [^14^C]acetate. The right image shows primuline staining of the same TLC plate. **(B)** Disintegration per minute (DPM) values of non-polar and polar lipids of the solvent control (ethanol) and fenpropimorph-treated cells (10 μg mL^-1^, 1 h, at 25°C) in TAP medium without acetate. Averages and SE from three replicate experiments are presented. Significant differences, as determined by Student’s *t*-test, are indicated by asterisks (**p < 0.01, ***p < 0.001).

If MGDG is an important source of FAs in TAG induced by the drug, it might be degraded to DAG, and then used for synthesis of TAG. To test this possibility, we labeled the cells with [^14^C]acetate, treated them with the drug, and detected radioactive TAG and DAG in control and drug-treated cells. The cells were labeled with [^14^C]acetate for 2 h, washed twice with TAP medium without acetate, and then treated with fenpropimorph for an additional hour before lipids were extracted. Total lipids were separated twice on a TLC plate, using two different solvent mixtures (the first for the separation of non-polar lipids, and the second for the separation of polar lipids). TAG spots were visible both under UV light (when stained with primuline; **Figure 5A**, right) and when using a radioisotope image analyzer (FLA-2000, Fujifilm; **Figure 5A**, left). DAG spots became visible only after 72 h or longer exposure of the TLC plate containing lipid extract from the fenpropimorph-treated cells, but not in that of mock-treated cells. (**Figure 5A**, left). We then compared the disintegration per minute (DPM) values of total non-polar lipids containing TAG, DAG, and free fatty acids with that of polar lipids, and found that the increase in DPM values of total non-polar lipids was equivalent to the decrease in that of the total polar lipids in cells subjected to fenpropimorph treatment (**Figure 5B**). Thus, the sum of DPM values of total lipids was similar between control and fenpropimorph-treated cells (**Figure 5B**), which again supports the notion that the fatty acids in fenpropimorph-induced TAGs originated from polar lipids.

### FENPROPIMORPH-INDUCED TAG ACCUMULATION IN *Chlamydomonas* IS NOT ACCOMPANIED BY CHANGES IN STEROL CONTENT OR COMPOSITION

**FIGURE 6:**
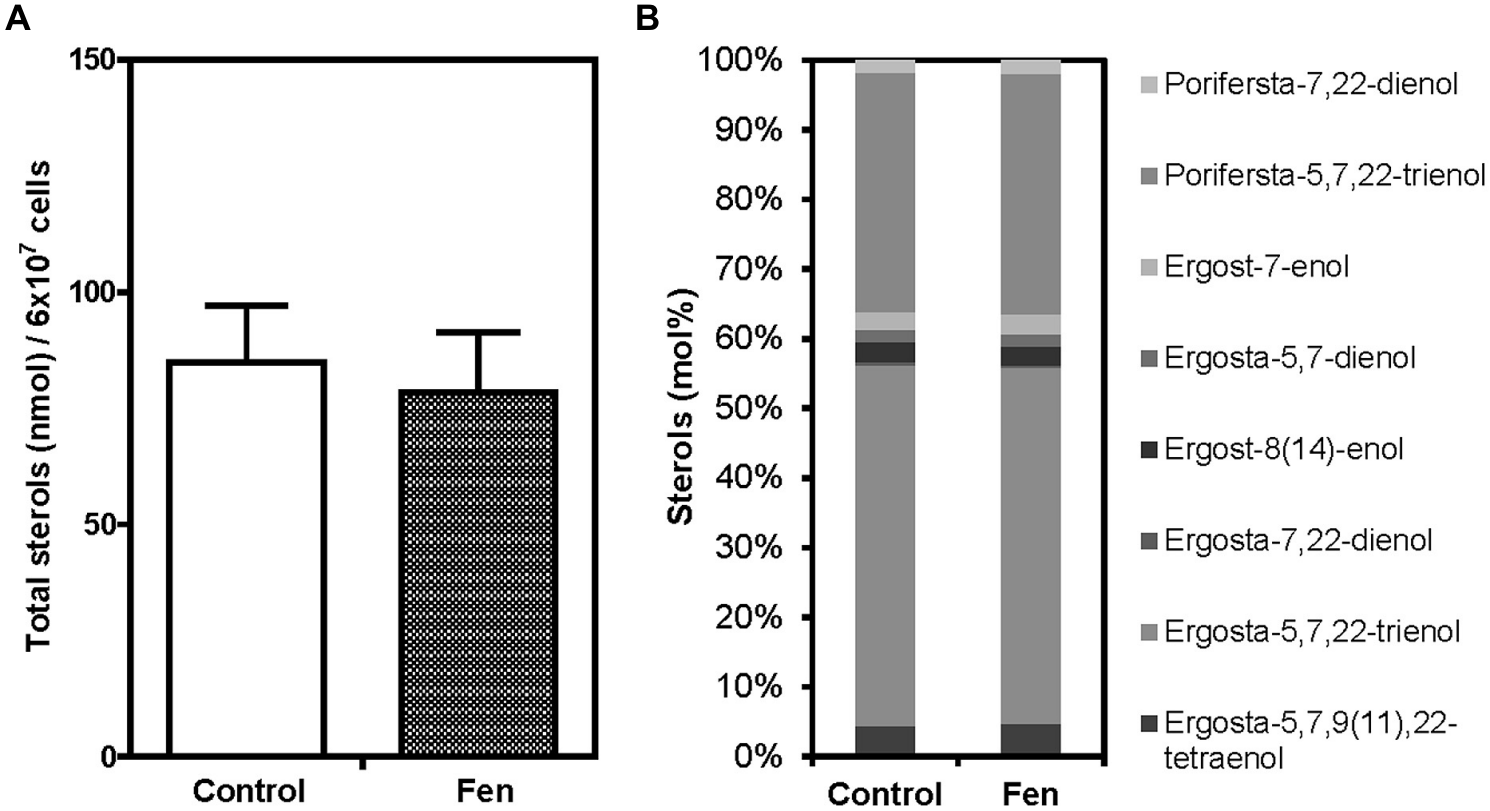
**Comparison of total sterol amount and sterol composition between control and fenpropimorph-treated *C. reinhardtii*. (A)** Total sterol amount of control and fenpropimorph-treated (10 μg mL^-1^, 1 h, at 25°C) cells. *Chlamydomonas* cells in late mid-log phase culture in TAP medium (N+, acetate+) were used. Averages from three replicate experiments are presented. Bars represent SE. **(B)** Sterol compositions of control and fenpropimorph-treated (10 μg mL^-1^, 1 h, at 25°C) cells.

Fenpropimorph treatment inhibits ergosterol biosynthesis in fungi (Baloch et al., 1984; Debieu et al., 1992; Moebius et al., 1996; Hartmann et al., 2002; Campagnac et al., 2009). To uncover the mechanism underlying fenpropimorph-induced TAG accumulation in *Chlamydomonas*, we tested whether fenpropimorph treatment changed the sterol levels or composition in this organism. Interestingly, neither the level of total sterols nor its composition changed in *Chlamydomonas* cells after 60 min of fenpropimorph treatment (**Figures 6A,B**).

### FENPROPIMORPH INDUCES SEVERE STRESS IN *Chlamydomonas* CELLS

**FIGURE A4:**
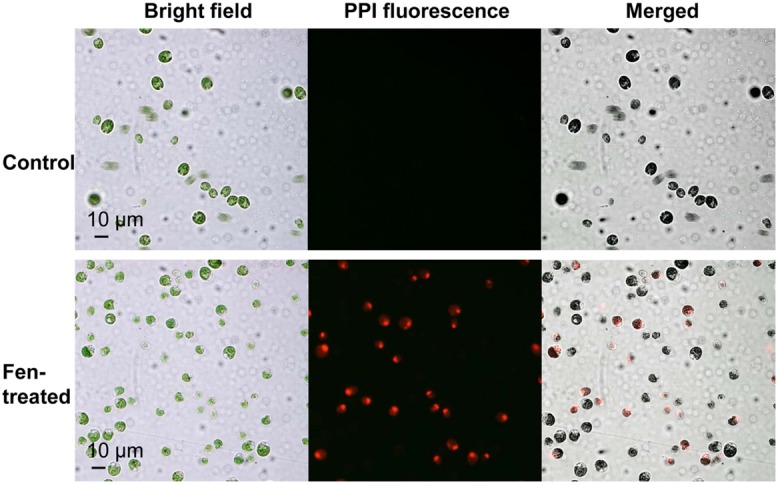
**Fenpropimorph treatment results in *Chlamydomonas* cell death.** Cells in TAP (N+, acetate+) medium were treated with fenpropimorph or ethanol (solvent control) for 1 h at 25°C, then stained with propidium iodide (2 μg mL^-1^, 15 min). Nuclear staining of cells treated with fenpropimorph suggests that the cells are dead.

**FIGURE A5:**
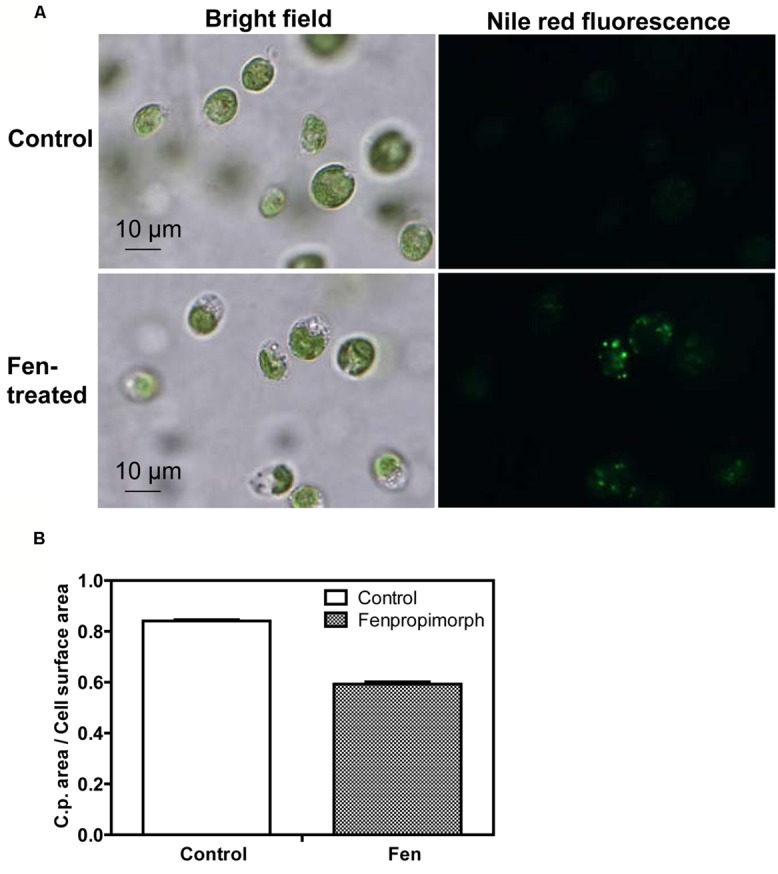
**Change in chloroplast morphology in fenpropimorph-treated *Chlamydomonas* cells. (A)** Chloroplasts are shrunken in fenpropimorph-treated *Chlamydomonas* cells. **(B)** Quantification of the chloroplast area per cell surface area in microphotographs of fenpropimorph-treated *Chlamydomonas* cells. Averages from fifty experimental replicates are presented. Bars represent SE.

To test whether fenpropimorph induces stress in the algal cells, we examined the motility of the treated cells. Immediately after treatment with fenpropimorph, most cells stopped moving, and after 1 h, most sank to the bottom of the container. Forty three percent of the cells (97 out of 224 cells counted) were dead as indicated by positive staining of the nucleus with propidium iodide at 1 h after treatment with fenpropimorph (**Figure A4**). This result suggests that fenpropimorph generated severe stress in the *Chlamydomonas* cells. Microscopy observations revealed that the plastids of fenpropimorph-treated cells were shrunken (**Figures 1C and A5**); the area of chloroplasts in fenpropimorph-treated cells was decreased by 29.5% in the treated cells (**Figure A5B**). This drastic change in chloroplast morphology was in agreement with the results of our lipid analysis: a large proportion of plastidial lipids degraded upon treatment with fenpropimorph.

## DISCUSSION

In this study, we found that fenpropimorph, a sterol biosynthesis inhibitor, rapidly (within an hour) induced at least a fourfold increase in the amount of TAGs produced by *C. reinhardtii* cells (**Figures 1 and 4**). We showed that fenpropimorph treatment induced the degradation of major chloroplastic lipids into fatty acids (**Figures 2 and 3**); recycling of the fatty acids to TAGs (**Figures 3 and 4**); and precipitation of TAG-induced cells at the end of culture. Previously reported stimuli that induce TAG, i.e., nutrient starvation, exposure to high light intensity, and salt stress, also degrade chloroplastic lipids and recycle the fatty acids to TAG (Fan et al., 2011; Siaut et al., 2011). However, striking difference exists between the previously reported stress-induced and fenpropimorph-induced TAG accumulation; it was very fast (**Figures 1 and 4**), the fastest among all triggers of algal lipid remodeling reported to date. We are not aware of any other treatment that induces such rapid remodeling of lipids as observed here with fenpropimorph treatment. Other triggers of lipid accumulation (nutrient starvation, high light intensity, and high salt) usually require several days.

**FIGURE A6:**
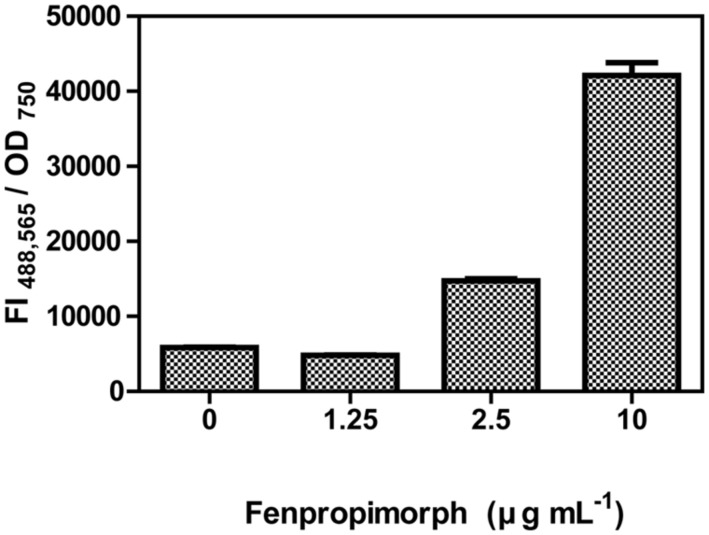
**Effect of 24-h treatment with various concentrations of fenpropimorph on Nile red fluorescence of *Chlamydomonas*.** Cells were incubated till late mid-log phase in the TAP (N+, acetate+) medium, treated with fenpropimorph (1.25, 2.5, or 10 μg mL^-1^) for 24 h at 25°C, then stained with Nile red. The fluorescence intensity (FI) was determined from three replicates and their averages are presented. Bars represent SE.

The level of total sterols or its composition did not change in *Chlamydomonas* cells which accumulated TAG upon fenpropimorph treatment (**Figures 6A,B**). The very short time required for TAG accumulation may have been too short for any changes in sterol metabolism, since, in other species, the drug induced change in sterol metabolism required days (Hartmann et al., 2002; Campagnac et al., 2008, 2009). Thus the mechanism underlying the rapid accumulation of TAG in fenpropimorph-treated *Chlamydomonas* cells does not seem to involve sterol metabolism. It remains to be determined whether fenpropimorph inhibits sterol synthesis in algae to the same extent as it does in fungi, and such a test would involve the long-term treatment of *Chlamydomonas* cells with a low dosage of the drug. We did not investigate further the effect of low concentrations of fenpropimorph, since up to 24 h treatment with 1.25 or 2.5 μg mL^-1^ of fenpropimorph did not induce high accumulation of TAG (**Figure A6**), but caused almost complete suppression of the growth of *Chlamydomonas.*

The plastidial origin of most of the fatty acids assimilated into TAGs in fenpropimorph-treated cells was concluded based on results from two experiments: fatty acid methyl ester (FAME) analysis (**Figure 2**), and membrane lipids analysis (**Figure 3C**). Upon the drug treatment, the levels of chloroplastic lipids, MGDG and PG decreased to half (**Figure 3C**). The TAG induced by the drug was much higher than that in control in 16:4(4,7,10,13) and 18:3(9,12,15) species of fatty acids (**Figure 2B**), which are found almost exclusively in chloroplast (Fan et al., 2011; Li et al., 2012). Chloroplast shrank upon the drug treatment (**Figure A5**), supporting the degradation of its internal lipids detected by biochemical analyses. These results and the kinetic analysis shown in **Figure 4** suggest that when the cells were exposed to fenpropimorph, MGDG was rapidly broken down within 10 min (**Figure 4B**), and the fatty acids or DAG liberated from MGDG and other plastidial lipids were used to synthesize TAG. The radioisotope analysis (**Figure 5**) indicated that the fatty acids liberated from MGDG and other plastidial lipids were transiently incorporated into DAG, and then used to synthesize TAG, because DAG was labeled in the fenpropimorph-treated cells. While the level of MGDG, the most abundant plastidial lipid, changed much upon the drug treatment, that of DGDG, another major plastidial lipid, did not change as much, similarly as found under nitrogen deficiency condition (Li et al., 2012). The reason for the different changes of the two plastidial lipids under nitrogen deficiency was attributed to the preference of MGDG over DGDG as substrate for PGD1, an enzyme critical for TAG accumulation under nitrogen deficiency (Li et al., 2012). Thus it is tempting to speculate that PGD1 may also be involved in fenpropimorph-induced TAG accumulation.

We previously reported that Brefeldin A (BFA), a drug that inhibits the intracellular trafficking of membranes, also induced TAG accumulation (Kim et al., 2013). Both BFA and fenpropimorph inhibit basic cellular functions, and this stress may be the common cause of TAG accumulation in cells treated with these chemicals. There is a difference and similarities between fenpropimorph-induced and BFA-induced TAG accumulation. A similarity is that both drugs induced lipid remodeling regardless of the carbon or nitrogen source in the medium. The striking differences between the two drug treatments include (i) fenpropimorph induced at least fourfold higher amount of TAG, whereas BFA induced maximum 1.3-fold higher amount of TAG, than their solvent controls (compare **Figure 1** of this paper with **Figures 2 and 3** of Kim et al., 2013); and (ii) the fatty acids in TAG induced by fenpropimorph were derived mostly from chloroplasts, whereas those induced by BFA treatment were from DGTS, a non-chloroplast membrane lipid (Kim et al., 2013). Thus, the two drugs function differently at the molecular level to induce TAG accumulation. Fenpropimorph might provide a useful tool in biodiesel production. Firstly, fenpropimorph treatment reduces the time for TAG induction. *Chlamydomonas* synthesizes TAGs under stress conditions, such as nutrient deficiency. Thus, a conventional method to induce TAG is to grow the cells under nutrient-rich conditions until they reach a certain density, and then to transfer the cells into nutrient-deficient medium to promote TAG accumulation. However, this process is time-consuming and expensive. In contrast to this conventional method, fenpropimorph treatment very rapidly induces TAG formation by converting polar lipids in the cell, suggesting that much time can be saved by using this drug. Moreover, the quantity of TAGs induced by 1 h treatment with fenpropimorph was much more than that produced by 9 days of nitrogen starvation in medium without acetate (**Figures 1E,F**). Under nitrogen replete condition, 10^7^ cells of *Chlamydomonas* contained about 10 μg fatty acid in TAG, while treatment with fenpropimorph increased it to about 50 μg. This level of TAG induced by fenpropimorph was higher than that found in the same number of cells of *Nannochloropsis* (25 μg), Chlorella (1 μg) or *Senedesmus* (8 μg) under normal condition (Siaut et al., 2011; Vigeolas et al., 2012; Simionato et al., 2013). Moreover, the drug may be able to increase TAG levels in many species of alga. Secondly, fenpropimorph treatment can improve the efficiency of biodiesel production, as it converts polar lipids into neutral lipids. Polar lipids such as phospholipids and galactolipids are usually removed during biodiesel production, since they inhibit the purification of TAGs by forming a gum layer, reducing the final amount of TAGs that can be used in biodiesel production (Watanabe et al., 2002; Lai et al., 2005). Thus, the *in vivo* reduction of polar lipids by fenpropimorph treatment might be advantageous to the biodiesel production process. Thirdly, fenpropimorph might provide an efficient method for harvesting the algal cells, since it induces cell death, and consequently, the precipitation of cells. Thus fenpropimorph might also reduce the cost in the harvesting step.

Thus we envisage that fenpropimorph can be used to cut down the cost of biodiesel production from the algae. If applied when the culture reaches at the maximum density, this chemical can save time and simplify steps to induce TAG accumulation, harvesting the cells, and purification of TAG. Further research into the molecular mechanism underlying fenpropimorph-induced TAG accumulation in *Chlamydomonas* might reveal yet other methods to rapidly accumulate TAG in algae for biodiesel production.

## Conflict of Interest Statement

The content of this paper was registered as a Korean patent 10-1428863.
